# Target Prediction for an Open Access Set of Compounds Active against *Mycobacterium tuberculosis*

**DOI:** 10.1371/journal.pcbi.1003253

**Published:** 2013-10-03

**Authors:** Francisco Martínez-Jiménez, George Papadatos, Lun Yang, Iain M. Wallace, Vinod Kumar, Ursula Pieper, Andrej Sali, James R. Brown, John P. Overington, Marc A. Marti-Renom

**Affiliations:** 1 Genome Biology Group, Centre Nacional d'Anàlisi Genòmica (CNAG), Barcelona, Spain; 2 Gene Regulation Stem Cells and Cancer Program, Centre for Genomic Regulation (CRG), Barcelona, Spain; 3 European Molecular Biology Laboratory – European Bioinformatics Institute (EMBL-EBI), Wellcome Trust Genome Campus, Hinxton, Cambridge, United Kingdom; 4 Computational Biology, Quantitative Sciences, GlaxoSmithKline, Collegeville, Pennsylvania, United States of America; 5 Department of Bioengineering and Therapeutic Sciences, University of California, San Francisco, San Francisco, California, United States of America; University of Maryland, Baltimore, United States of America

## Abstract

*Mycobacterium tuberculosis*, the causative agent of tuberculosis (TB), infects an estimated two billion people worldwide and is the leading cause of mortality due to infectious disease. The development of new anti-TB therapeutics is required, because of the emergence of multi-drug resistance strains as well as co-infection with other pathogens, especially HIV. Recently, the pharmaceutical company GlaxoSmithKline published the results of a high-throughput screen (HTS) of their two million compound library for anti-mycobacterial phenotypes. The screen revealed 776 compounds with significant activity against the *M. tuberculosis* H37Rv strain, including a subset of 177 prioritized compounds with high potency and low *in vitro* cytotoxicity. The next major challenge is the identification of the target proteins. Here, we use a computational approach that integrates historical bioassay data, chemical properties and structural comparisons of selected compounds to propose their potential targets in *M. tuberculosis*. We predicted 139 target - compound links, providing a necessary basis for further studies to characterize the mode of action of these compounds. The results from our analysis, including the predicted structural models, are available to the wider scientific community in the open source mode, to encourage further development of novel TB therapeutics.

## Author Summary

*Mycobacterium tuberculosis* is a major worldwide pathogen infecting millions individuals every year. Additionally, the number of antibiotic resistant strains has dramatically increased over the last decades. Trying to address this challenge, the pharmaceutical company GlaxoSmithKline has recently published the results of a large-scale high-throughput screen (HTS) that resulted in the release of 776 chemical compound structures active against tuberculosis. We have used this dataset of compounds as input to our computational approach that integrates historical bioassay data, chemical properties and structural comparisons. We propose 139 targets alongside their respective hit compounds and made them open to the wider scientific community. Our hope is that the availability of the experimental data from GSK and our computational analysis will encourage further research providing validated therapeutically targets against this devastating disease.

## Introduction

One third of the world's population is infected with *Mycobacterium tuberculosis* (MTB), the causative agent of tuberculosis [1]. Approximately 95% of infected individuals are thought to have persistent, latent MTB infections that remain dormant until activated by specific environmental and host response events. Approximately 10% of latent infections eventually progress to active disease, which, if left untreated, kills more than half of the infected patients [2]. Moreover, there is an increasing clinical occurrence of MTB strains with extensive multi-drug-resistance (*eg*, MTB MDR and MTB XDR), where mortality rates can approach 100% [3]. In some countries, the MTB MDR and XDR strains may account for up to 22% of infections [1]. In addition, current TB therapeutic regimes involve a combination of antibiotics, administered at regular intervals over a 6-month period, which makes patient compliance an issue, especially in developing countries [1], [2].

The discovery and development of new antibiotics is widely recognized as one of the major global health emergencies, yet it is also a major pharmaceutical challenge. Most currently used antibiotics were discovered during the golden era from the 1940s to 1960s through large scale screening of compound collections for anti-bacterial activity – the so-called whole cell or phenotypic screens [4]. The emergence of bacterial molecular genomics technologies and the availability of whole genome sequences in the 1990s led to dramatic changes in anti-bacterial drug discovery, where the emphasis was placed on screening essential targets for inhibitory compounds. However, despite intensive efforts, target-based screening has been largely unsuccessful in producing clinical candidate molecules [5]. As a result, a return to whole cell screening has been widely advocated, in combination with novel technologies and bioinformatics to rapid identify targets associated with a compound's mechanism of action (MOA) [4], [6].

Recently, the pharmaceutical company GlaxoSmithKline (GSK) completed an anti-mycobacterial phenotypic screening campaign against *M. bovis* BCG, a non-virulent, vaccine *Mycobacterium* strain, with a subsequent secondary screening in *M. tuberculosis* H37Rv (MTB H37Rv) for hit confirmation [7]. A total of 776 potent compound hits (including 177 MTB H37RV hits with limited human cell line toxicity) were made openly available to the wider scientific community through the ChEMBL database (http://dx.doi.org/10.6019/CHEMBL2095176). The aim of this release was to stimulate mechanism of action analyses using chemical genetics/proteomics approaches, as well as to provide many potential new starting points for synthetic lead generation activities. To attain these goals, it is essential to identify the likely protein targets of these active compounds. Here, we introduce an integrative computational analysis towards the genome-wide characterization of targets for selected compounds against tuberculosis. Our approach is in contrast to the classical target-based experiments, widely used in drug discovery, that suffer from very high attrition rates in anti-infective molecules [8]. This study should also serve the wider anti-tuberculosis research community by providing a list of genes and pathways that are more likely to be validated as TB targets for drug discovery and development.

We applied computational approaches using three domains of knowledge, namely the “assay space”, “chemogenomics space” and “structural space”, to identify new targets that are likely to interact with the active compounds from the GSK collection. We characterized the structural and chemical spaces of the recently released set of 776 compounds active against tuberculosis [7] and grouped the compounds into a total of 551 structural families. Subsequently, we predicted their likely targets using three orthogonal and complementary computational approaches. Jointly, we identified several amino-acid biosynthesis proteins as possible targets of several compounds in the dataset. A total of 207 unique pairs of compounds and potential MTB targets have been predicted. These compounds constitute a basis for further hypothesis-led exploration of their mode of action. We briefly outline the possible impact and contribution of our findings to Open Drug Discovery Initiatives [9], [10], [11], in particular against tuberculosis.

## Results/Discussion

### The TCAMS-TB compound dataset

**Figure 1:**
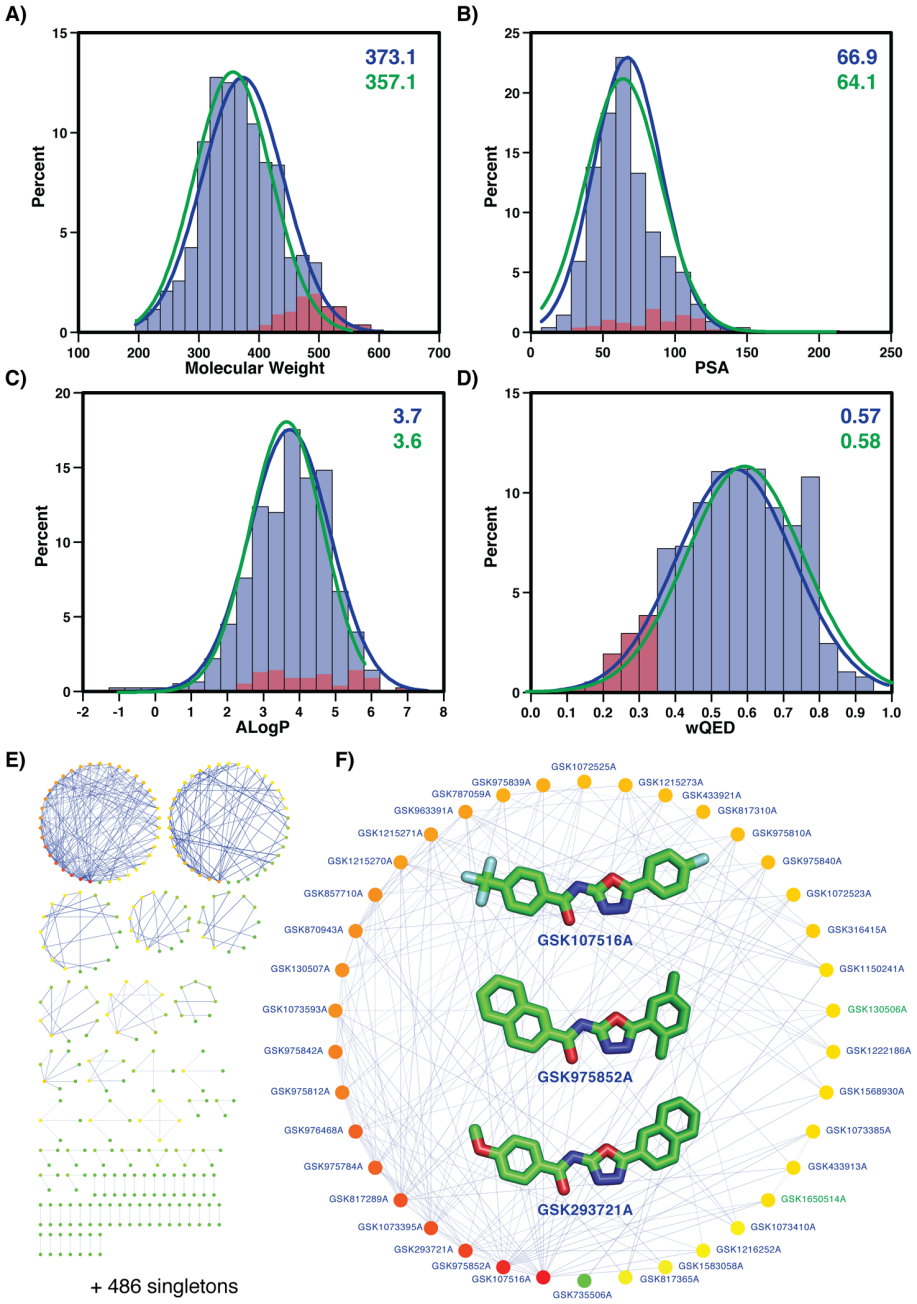
GSK dataset of 776 compounds. Panels A to D describe the drug-like properties of the compounds, including the subset of 177 compounds active against MTB (green color). Red colored subsets correspond to compounds with weighted QED score smaller than 0.35 [12]. The distribution's mean values are shown in the top-right corner of each plot. **A**) Molecular weight distribution. **B**) PSA distribution. **C**) ALogP distribution. **D**) Weighted QED distribution. Panels E and F show the structural clusters of the compounds. Links between compounds indicate 0.9 or higher RFS similarity. **E**) Entire network of 776 compounds resulting in 551 structural families (486 singletons). **F**) Highlight of family number 1 with 38 compounds (inner images for the three most connected compounds in the family).

GSK recently released the data from a phenotypic screen against tuberculosis (available at ChEMBL http://dx.doi.org/10.6019/CHEMBL2095176) [7]. This open access dataset contains a total of 776 compounds active against *M. bovis* BCG, a non-virulent *Mycobacterium* species widely used in experimental studies as a vaccine component, and a subset of 177 confirmed compounds active against MTB strain H37Rv. The compound collection had been pre-filtered to remove known anti-bacterial compounds to maximize the discovery of novel compounds with anti-Mycobacterium activities. About 90% of the compounds have a quantitative estimate of drug-likeness (QED) value above 0.35 [12], herein called optimal drug-like compounds (Figure 1). The remaining 10% of compounds, which are highlighted by red bars in Figure 1, have higher molecular weights (>400 KD) and slightly higher hydrophobicity, expressed as the calculated logarithm of the 1-octanol/water partition coefficient (ALogP) [13]. For the subset of 177 compounds active against H37Rv, the average molecular weight is statistically smaller than for the entire dataset (Figure 1), consistent with known trends of lipophilicity and cytotoxicity/polypharmacology. The molecular PSA (polar surface area), ALogP (octanol–water partition coefficient) and wQED (weighted QED) scores result in statistically indistinguishable average values and distributions for both datasets. To assess the diversity of the dataset, we applied our Random Forest Score (RFS) to identify pairs of similar compounds (Methods). An all-against-all comparison was performed by nAnnolyze [14] and any pair of compounds with an RFS higher than 0.9 were considered similar. The resulting network of compound similarities was layered using Cytoscape [15] (Figure 1E). The entire dataset of 776 compounds was clustered into a total of 551 compound families, primarily composed of two large compound families and 481 singleton families. The two large families of compounds (GSKFAM_1 and GSKFAM_2) included 38 compounds each connected by 156 and 80 links, respectively (Figure 1F). In summary, the active compound set released by GSK is composed of drug-like molecules with non-redundant and diverse scaffolds.

### Integrative computational analysis

The 776 compounds released by GSK were used as input to our integrative computational analysis approach that combines the results from a chemogenomics space search (CHEM), a structural space search (STR) and a historical assay space search (HIST). First, the exploration of the chemical space allowed us to identify likely targets for the input compounds based on their structural similarity to compounds with experimentally validated targets deposited in the ChEMBL database [16]. The approach employed a multi-category Naïve Bayesian classifier, which has been successfully used in ligand-based target prediction efforts [17], [18], [19]. Second, the exploration of the structural space allowed for the identification of likely targets based on the structural similarity of compounds and protein targets with known three-dimensional structures. The method was based on an improved version of the AnnoLyze program [14]. Finally, the exploration of the historical data on screening assays resulted in testable hypotheses for the anti-*Mycobacterium* mode of action of the selected compounds, based on the historical data from internal GSK screening experiments. This integrative approach allowed us to predict targets for the set of released compounds in the absence of known structural data (CHEM and HIST) or the absence of knowledge of the binding site (STR). When the three-dimensional structure of the target and the localization of the binding site are known or predicted, it is often helpful to follow up with molecular docking (see [20] and examples below). However, such an approach would be prohibitive for large numbers of compounds against a large number of targets, because molecular docking results still need to be interpreted manually for best impact. The three methods used in our integrative approach are further detailed in the Methods section of this manuscript.

### Chemogenomics space (CHEM)

We applied a multi-category Naïve Bayesian classifier (MCNBC) that was built and trained using structural and bioactivity information from the ChEMBL database [16]. Given a new compound, the model calculates a likelihood score based on the molecule's individual sub-structural/fingerprint features and produces a ranked list of likely targets. In total, the 776 compounds in the *M. bovis* BCG dataset resulted in 2,179 statistically significant target associations (at a Z-score >2.0) to proteins in the ChEMBL database from 62 different organisms (63% of hits are to human proteins). A simple orthology search against the MTB proteins from this set resulted in 1,401 compound-target relationships for 84 MTB proteins, with detectable orthology to 34 organisms. The specific predictions from the chemical space search are available at http://www.tropicaldisease.org/TCAMSTB (CHEM type).

### Structural space (STR)

We applied a Random Forest Score that identified structural similarities between any compound in the dataset and ligands from the Protein Data Bank (PDB) [14]. Each compound in the *M. bovis* BCG dataset is compared to ∼2,500 ligands for which there are known complex structures in the PDB, identifying structural similarities to be included in a pre-built network of structural relationships between ligands and targets. In total, the 776 compounds resulted in 207 significant target associations (RFS score >0.4) to proteins in a set of modeled three-dimensional structures from the MTB proteome. The specific predictions from the structural space search are available at http://www.tropicaldisease.org/TCAMSTB (STR type).

### Historical assay space (HIST)

We used the historical GSK bioassay data to develop hypotheses for the anti-*Mycobacterium* mode of action for the active compounds. Using conservative activity thresholds, we found among the compounds active against MTB H37Rv unambiguous annotations for 49 compounds and their previously measured activity in 120 biochemical assays against 63 human targets (*i.e.*, sub-micromolar IC50 or EC50). Overall, the *M. bovis* BCG screens resulted in a considerably larger number of active compounds and thus have a correspondingly greater amount of historical assay information. A total of 240 compounds were found to have activity recorded in 642 assays involving 209 human targets, with the largest human target classes being GPCRs and protein kinases, as expected. We then searched for orthologous sequences of the human assayed proteins in the MTB H37Rv and *M. bovis* BCG genomes using conservative criteria for assigning human-*Mycobacterium* homology (BLAST E-value ≤1.0e^−10^). Although there are significant evolutionary differences between bacterial and mammalian genomes, we still found 19 *M. bovis* BCG homologous genes (Table S1) in different target classes (Figure S1), including kinases (8 genes), cytochrome P450s (2 genes) and nine other enzymes such as a putative D-amino acid oxidase, an amidase, a putative flavin-containing monoamine oxidase, a NAD-dependent deacetylase, a putative catechol-O-methyltransferase, a protease, a putative epoxide hydrolase, a 3-ketoacyl-(acyl-carrier-protein) reductase, and a dihydroorotate dehydrogenase 2. While these *M. bovis* BCG genes had orthologous sequences in MTB H37Rv, fewer compounds were associated with putative targets in the latter species. For example, two *Mycobacterium* kinases and five enzymes were exclusively associated with *M. bovis* BCG positive compounds. Two kinases (pknA and pknB) and one enzyme (fabG) were experimentally characterized as essential for the survival of MTB [21], [22]. A total of 20 and 94 compounds were indirectly mapped by human protein target homology to 12 MTB H37Rv and 19 *M. bovis* BCG genes, respectively. The specific predictions from the historical assay space search are detailed in Supporting Information and are available at http://www.tropicaldisease.org/TCAMSTB (HIST type).

### Subset of compounds with predicted targets

**Figure 2:**
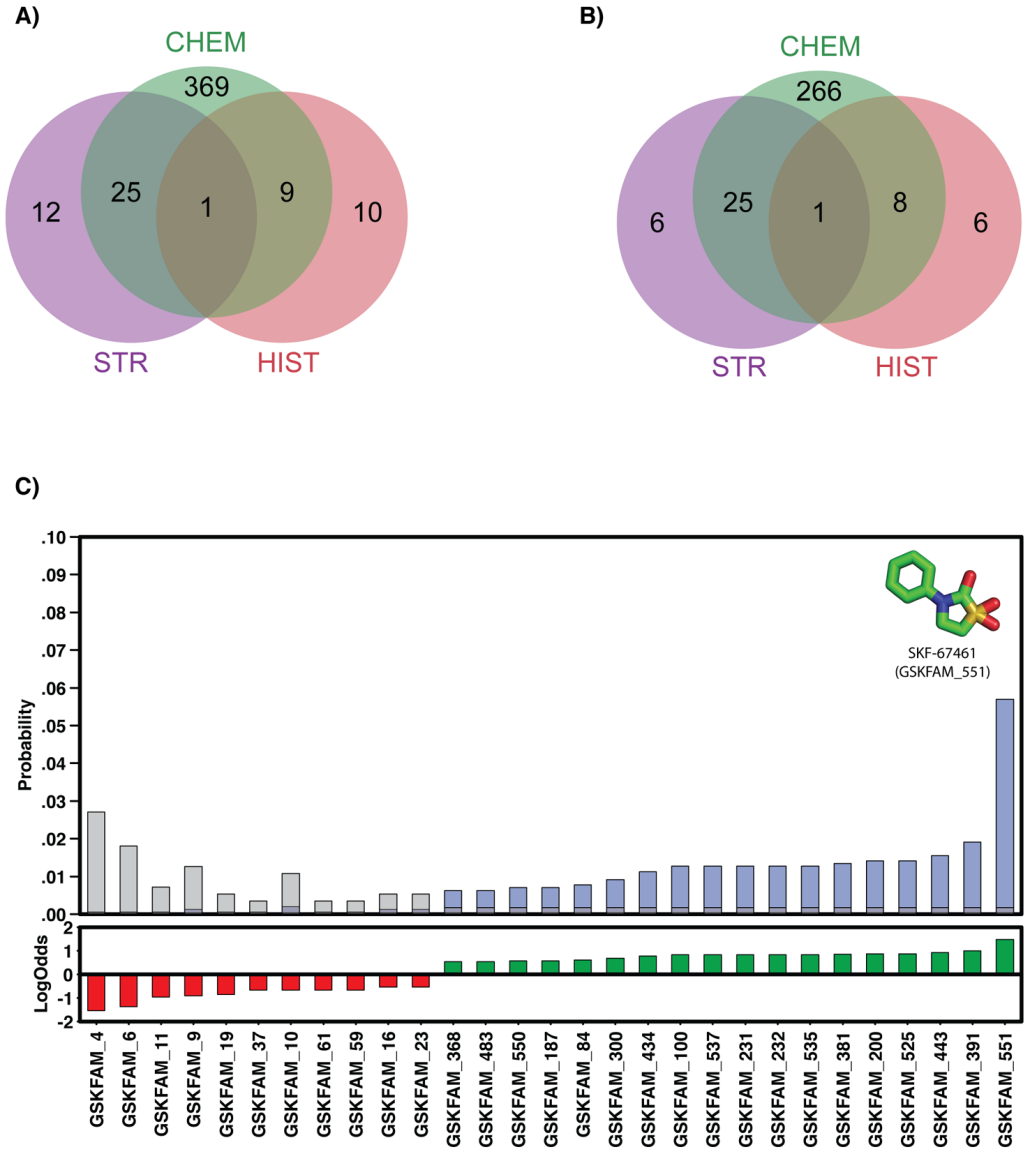
Subset of GSK compounds with predicted targets. **A**) Venn diagram with common compounds with predictions from the three different approaches (that is, in green from the search of the chemogenomics space, in purple from the search of the structural space, and in red from the historical data). **B**) Venn diagram with common compound families with predictions from the three different approaches. **C**) Most under and over-represented chemical families in our predictions. Upper plot shows the probability of finding a given family in the original dataset (grey bars) compared to the probability of finding it in the dataset with predicted targets (blue bars). Lower plot shows the log odds per selected family (*i.e.*, absolute log odds larger than 0.5).

Of the 776 compounds in the GSK dataset, only one compound (GSK445886A) was predicted to hit diverse targets from different pathways by the three independent methods (Figure 2A). A total of 25 and 9 compounds were jointly predicted to hit a target by CHEM/STR and CHEM/HIST searches, respectively. The majority of predictions were obtained by the CHEM approach (404 compounds with predicted targets), followed by the STR approach (38 compounds with a predicted target) and the HIST approach (20 compounds with predicted targets). Such results were expected because the available information on biological activity shrinks as we move from the general “chemical” to the more specific “structural” and “historical” spaces. Interestingly, as an indication of the orthogonality of the three approaches, most of the redundancy of compounds with a predicted target was specific to each approach. In other words, each of the three approaches covered different parts of the space of compound-target predictions. For example, the CHEM approach predicted a target for 300 compound families (compared to a total of 404 unique compounds), of which it still shared 34 with either the STR or the HIST approaches (Figure 1B). A similar trend was observed for the other two approaches, indicating that the common compounds mostly occurred in small compound families or even singletons. Indeed, the GSK445886A compound, which was predicted to have a target by all three approaches, corresponded to a singleton compound family (GSKFAM_293).

To identify whether the three different approaches predicted targets for specific families in the dataset, we calculated the log odds probability (LogOdd) of a given compound family to appear in the list of selected compounds, given their different distributions in the original dataset (Figure 2C). This analysis aimed at identifying possible biases or artifacts specific to each of the three independent methods used in our integrative approach. Eleven compound families were under-represented in the selected dataset and 18 families were over-represented (with LogOdd values smaller than −0.5 and greater than 0.5, respectively). Interestingly, GSKFAM_551, which is a singleton with the SKF-67461 compound, was over-represented in the subset of selected compounds. Such predictions were based mostly on the STR and CHEM searches and may correspond to the chemical properties of the compound, resulting in a high false-positive rate for those two approaches. Conversely, the GSKFAM_4, which contains 15 compounds, is under-represented in the final subset of selected compounds, with only 1 hit identified by the CHEM approach.

### Predicted targets

**Figure 3:**
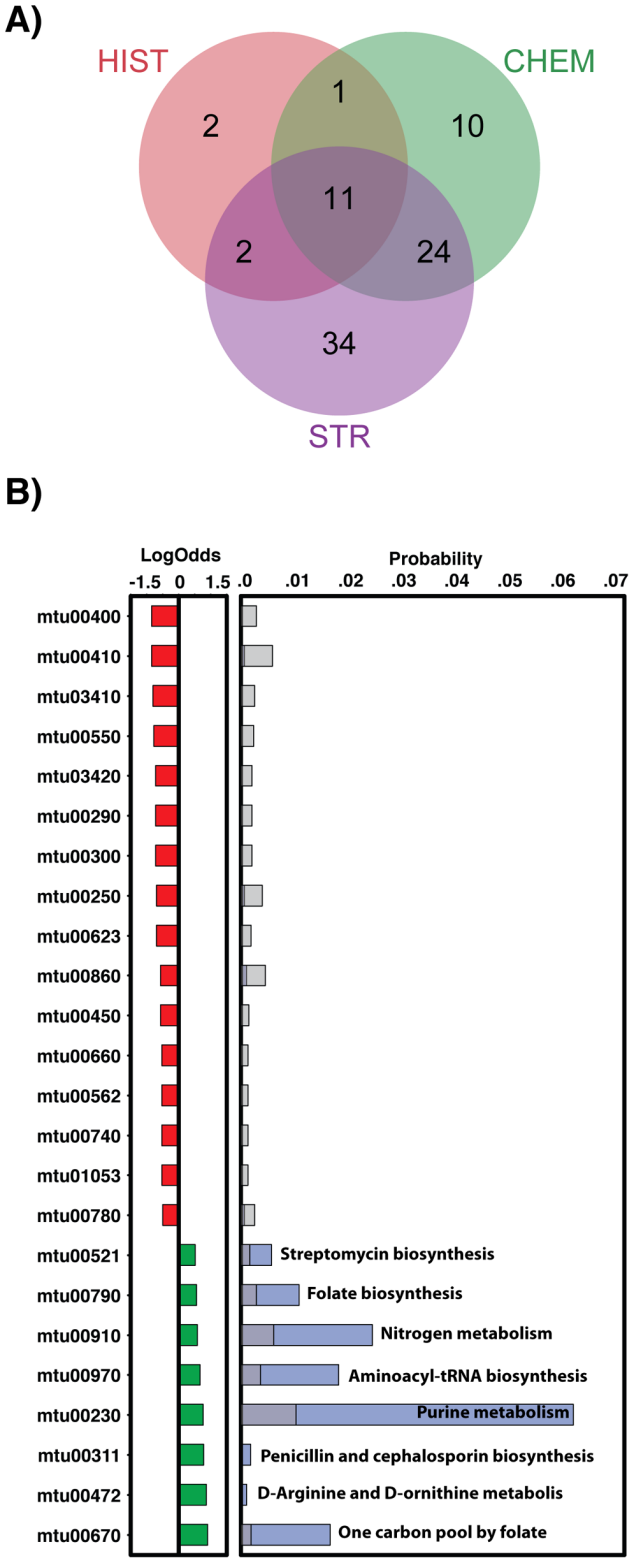
Predicted KEGG pathways targeted by the GSK compounds. **A**) Venn diagram with common pathways from the three different approaches. **B**) Most under and over-represented pathways in our predictions. Panels A) and B) with the same representation as in Figure 2.

There are a total of 1,044 unique MTB targets associated with a total of 112 pathways annotated in the KEGG database [23] (the mtu identifiers below refer to the relevant KEGG pathway id). Of those, the three orthogonal approaches identified targets for the selected set of compounds in a total of 84 pathways (Figure 3A). The STR search resulted in hits to 71 unique pathways, while the CHEM and the HIST searches resulted in hits to 35 and 16 pathways, respectively. These results were expected, because the target information is reduced from the STR space to the HIST space. A total of 11 unique pathways were predicted by the three approaches (Figure 3A and Table 1); these include many pathways associated with amino acid and nucleotide metabolism, such as arginine and proline metabolism (mtu00330), tryptophan metabolism (mtu00380), phenylalanine metabolism (mtu00360), tyrosine metabolism (mtu00350), histidine metabolism (mtu00340), glycine/serine/threonine metabolism (mtu00260) and pyrimidine metabolism (mtu00240). The results indicate that the GSK compounds potentially target proteins associated with primary metabolism. Interestingly, another seven pathways, not identified by the HIST approach, were found over-represented in the final set of predicted targets (Figure 3B). Those include some further primary and secondary metabolism systems, including streptomycin biosynthesis (mtu00521), folate biosynthesis (mtu00790), nitrogen metabolism (mtu00910), aminoacyl-tRNA biosynthesis (mtu00970), purine metabolism (mtu00230), penicillin and cephalosporin biosynthesis (mtu00311), D-arginine and D-ornithine metabolism (mtu00472), and one carbon pool by folate (mtu00670).

**Table 1:** List of seven common hit pathways identified by the three independent approaches.

Pathway	Approach	Targets	Compound families
**mtu00240**	STR	*Rv1381*	**255**
**Pyrimidine metabolism**		Rv3048c	**86**
****		Rv3314c	**255**
	CHEM	Rv2139	**Several**
		Rv2764c	**Several**
		Rv3247c	**497**
	HIST	Rv2139	**2**
**mtu00260**	STR	Rv0489	**551**
**Glycine, serine and threonine metabolism**		*Rv1296*	**551**
		*Rv3708c*	**551**
	CHEM	Rv1905c	**5,252,497**
		Rv3170	**Several**
	HIST	Rv3170	**5**
**mtu00330**	STR	*Rv1652*	**476,488**
**Arginine and proline metabolism**	CHEM	Rv0458	**60**
		Rv1905c	**5,252,497**
		Rv3170	**Several**
	HIST	Rv1263	**5,272**
		Rv3170	**5**
**mtu00340**	STR	Rv0187	**551**
**Histidine metabolism**		Rv0520	**551**
		Rv1498c	**300**
		Rv1603	**551**
		Rv1605	**551**
	CHEM	Rv0458	**60**
		Rv3170	**Several**
	HIST	Rv3170	**5**
**mtu00350**	STR	Rv0187	**551**
**Tyrosine metabolism**		Rv0520	**551**
		Rv1498c	**300**
		Rv1703c	**551**
	CHEM	Rv3170	**Several**
	HIST	Rv3170	**5**
**mtu00360**	STR	Rv1908c	**551**
**Phenylalanine metabolism**		Rv3469c	**551**
	CHEM	Rv3170	**Several**
	HIST	Rv1263	**5,272**
		Rv3170	**5**
**mtu00380**	STR	Rv0859	**551**
**Tryptophan metabolism**		Rv1908c	**551**
	CHEM	Rv0458	**60**
		*Rv1323*	**Several**
		Rv3170	**Several**
	HIST	Rv1263	**5,272**
		Rv3170	**5**

The additional four common pathways identified not shown correspond to general pathway descriptions (i.e., mtu01100 “Metabolic pathways”, mtu01110 “Biosynthesis of secondary metabolites”, mtu01120 “Microbial metabolism in diverse environments”, and mtu00000 “No Pathway”). Target genes in italics are either *in vivo* or *in vitro* essential in the TraSH Essentiality database [21].

### Predicted pairs of compound-target

To assess the significance of our predictions using the three different approaches, we calculated a t-statistics p-value of any compound family - KEGG pathway pair (Methods). The search identified 8 different compound families with significant links (p-value <1×10^−5^) to 14 different KEGG pathways (Table 2). The GSK compound family 1, through its compounds GSK975784A, GSK975810A, GSK975839A, GSK975840A and GSK975842A, was predicted to target the glycerolipid (mtu00561) and glycerophospholipid metabolisms (mtu00564), with significant over-representation through 6 different targets including Rv2182c and Rv2483c, both acyltransferases essential for the survival of the bacteria [21]. The GSK compound family 3 was predicted to target the ABC transporters (mtu02010) through its compounds GSK547481A, GSK547490A, GSK547491A, GSK547499A, GSK547500A, GSK547511A, GSK547512A, GSK547527A, GSK547528A and GSK547543A. Similarly, it was also predicted to target the aminoacyl-tRNA biosynthesis (mtu00970) pathways, through 3 different targets including Rv1640c, a lysyl-tRNA synthetase essential for the survival of the bacteria [21]. The GSK compound family 7, was predicted to target several pathways through 2 different targets Rv0053 (30S ribosomal protein S6) and Rv0650 (a glucokinase), none considered essential for the survival of the bacteria [21]. The GSK compound family 9 through its compounds GSK1188379A and GSK1188380A, was predicted to target the ABC transporters (mtu02010) pathway through the Rv0194 target (ATP-binding cassette, subfamily C) considered non-essential for the survival of the bacteria [21]. Identical results were obtained with the GSK compound family 16 through its compounds GSK1825940A and GSK1825944A. The GSK compound family 35 through its compounds BRL-10143SA and BRL-51093AA was predicted to target the one carbon pool by folate (mtu00670) pathway through the Rv2763c and Rv2764c targets (a dihydrofolate reductase and a thymidylate synthase, respectively) considered non-essential for the survival of the bacteria [21]. The GSK compound family 173 through its compound GSK14022909A was predicted to target the aminoacyl-tRNA biosynthesis (mtu00970) pathway through three essential targets [21], Rv1640c, Rv3598c and Rv3834c (a lysyl-tRNA synthetase, a lysyl-tRNA ligase, and a seryl-tRNA ligase, respectively), which are essential for the survival of the organism [21]. Interestingly, this family is also predicted to target Rv3105c and Rv3135 genes (a peptide chain release factor 2 and a PPE family protein), which are also essential for the survival of the organism [21]. Finally, the GSK compound family 334 through compound GSK270671A was predicted to target the nitrogen metabolism (mtu00910) pathway through the Rv1284 and Rv3588 targets (carbonic anhydrases) considered essential for the survival of the bacteria [21].

**Table 2:** Significant links between GSK compound families and KEGG pathways.

GSK Family	Compound	Target	Pathways
1	GSK975784A	*Rv2182c*	**Glycerolipid metabolism (mtu00561)**
			**Glycerophospholipid metabolism (mtu00564)**
		*Rv2483c*	No Pathway
	GSK975810A	*Rv2182c*	**Glycerolipid metabolism (mtu00561)**
			**Glycerophospholipid metabolism (mtu00564)**
		*Rv2483c*	No Pathway
	GSK975839A	*Rv2182c*	**Glycerolipid metabolism (mtu00561)**
			**Glycerophospholipid metabolism (mtu00564)**
		*Rv2483c*	No Pathway
		Rv2299c	No Pathway
	GSK975840A	*Rv2182c*	**Glycerolipid metabolism (mtu00561)**
			**Glycerophospholipid metabolism (mtu00564)**
		*Rv2483c*	No Pathway
	GSK975842A	*Rv2182c*	**Glycerolipid metabolism (mtu00561)**
			**Glycerophospholipid metabolism (mtu00564)**
		*Rv2483c*	No Pathway
		Rv2045c	No Pathway
		Rv2139	Pyrimidine metabolism (mtu00240)
		Rv2299c	No Pathway
		Rv2483c	No Pathway
3	GSK547481A	Rv0194	**ABC transporters (mtu02010)**
	GSK547490A	Rv0194	**ABC transporters (mtu02010)**
	GSK547491A	Rv0194	**ABC transporters (mtu02010)**
	GSK547499A	Rv0194	**ABC transporters (mtu02010)**
	GSK547500A	Rv0194	**ABC transporters (mtu02010)**
	GSK547511A	Rv0194	**ABC transporters (mtu02010)**
	GSK547512A	Rv0194	**ABC transporters (mtu02010)**
	GSK547527A	*Rv1640c*	**Aminoacyl-tRNA biosynthesis (mtu00970)**
		Rv3598c	**Aminoacyl-tRNA biosynthesis (mtu00970)**
		Rv0194	**ABC transporters (mtu02010)**
	GSK547528A	*Rv1640c*	**Aminoacyl-tRNA biosynthesis (mtu00970)**
		Rv3598c	**Aminoacyl-tRNA biosynthesis (mtu00970)**
		Rv0194	**ABC transporters (mtu02010)**
	GSK547543A	Rv0194	**ABC transporters (mtu02010)**
7	GSK1829727A	Rv0053	**Ribosome (mtu03010)**
		Rv0379	No Pathway
		Rv0650	Glycolysis/Gluconeogenesis (mtu00010)
			**Galactose metabolism (mtu00052)**
			**Starch and sucrose metabolism (mtu00500)**
			**Amino sugar & nucl. sugar metab. (mtu00520)**
			**Streptomycin biosynthesis (mtu00521)**
	GSK1829729A	*Rv3855*	No Pathway
		Rv0053	**Ribosome (mtu03010)**
		Rv0379	No Pathway
		Rv0650	Glycolysis/Gluconeogenesis (mtu00010)
			**Galactose metabolism (mtu00052)**
			**Starch and sucrose metabolism (mtu00500)**
			**Amino sugar & nucl. sugar metab. (mtu00520)**
			**Streptomycin biosynthesis (mtu00521)**
	GSK1829816A	Rv0053	**Ribosome (mtu03010)**
		Rv0379	No Pathway
		Rv0650	Glycolysis/Gluconeogenesis (mtu00010)
			**Galactose metabolism (mtu00052)**
			**Starch and sucrose metabolism (mtu00500)**
			**Amino sugar & nucl. sugar metab. (mtu00520)**
			**Streptomycin biosynthesis (mtu00521)**
	GSK479031A	Rv0053	**Ribosome (mtu03010)**
		Rv0379	NoPathway (mtu00000)
		Rv0650	Glycolysis/Gluconeogenesis (mtu00010)
			**Galactose metabolism (mtu00052)**
			**Starch and sucrose metabolism (mtu00500)**
			**Amino sugar & nucl. sugar metab. (mtu00520)**
			**Streptomycin biosynthesis (mtu00521)**
	GSK957094A	Rv3170	Gly, Ser and Thr metabolism (mtu00260)
			Arginine and proline metabolism (mtu00330)
			Histidine metabolism (mtu00340)
			Tyrosine metabolism (mtu00350)
			Phenylalanine metabolism (mtu00360)
			Tryptophan metabolism (mtu00380)
		Rv0053	**Ribosome (mtu03010)**
		Rv0379	No Pathway
		Rv0650	Glycolysis/Gluconeogenesis (mtu00010)
			**Galactose metabolism (mtu00052)**
			**Starch and sucrose metabolism (mtu00500)**
			**Amino sugar & nucl. sugar metab. (mtu00520)**
			**Streptomycin biosynthesis (mtu00521)**
9	GSK1188379A	Rv0194	**ABC transporters (mtu02010)**
	GSK1188380A	Rv0194	**ABC transporters (mtu02010)**
16	GSK1825940A	Rv0194	**ABC transporters (mtu02010)**
	GSK1825944A	Rv0194	**ABC transporters (mtu02010)**
35	BRL-10143SA	*Rv1649*	Aminoacyl-tRNA biosynthesis (mtu00970)
		Rv2763c	**One carbon pool by folate (mtu00670)**
			Folate biosynthesis (mtu00790)
			**One carbon pool by folate (mtu00670)**
		Rv2764c	Pyrimidine metabolism (mtu00240)
	BRL-51093AM	Rv2763c	**One carbon pool by folate (mtu00670)**
		Rv2764c	Folate biosynthesis (mtu00790)
			**One carbon pool by folate (mtu00670)**
			Pyrimidine metabolism (mtu00240)
173	GSK1402290A	*Rv1640c*	**Aminoacyl-tRNA biosynthesis (mtu00970)**
		*Rv3598c*	**Aminoacyl-tRNA biosynthesis (mtu00970)**
		*Rv3834c*	**Aminoacyl-tRNA biosynthesis (mtu00970)**
		*Rv3105c*	No Pathway
		*Rv3135*	No Pathway
334	GSK270671A	*Rv1284*	**Nitrogen metabolism (mtu00910)**
		*Rv3588c*	**Nitrogen metabolism (mtu00910)**
		Rv3273	**Nitrogen metabolism (mtu00910)**
		Rv1707	No Pathway

Target genes in italics are either *in vivo* or *in vitro* essential in the TraSH Essentiality database [21]. Pathways highlighted in bold are responsible of the significant link to the GSK family.

### An example of a serine/threonine-protein kinase (pknB) target

**Figure 4:**
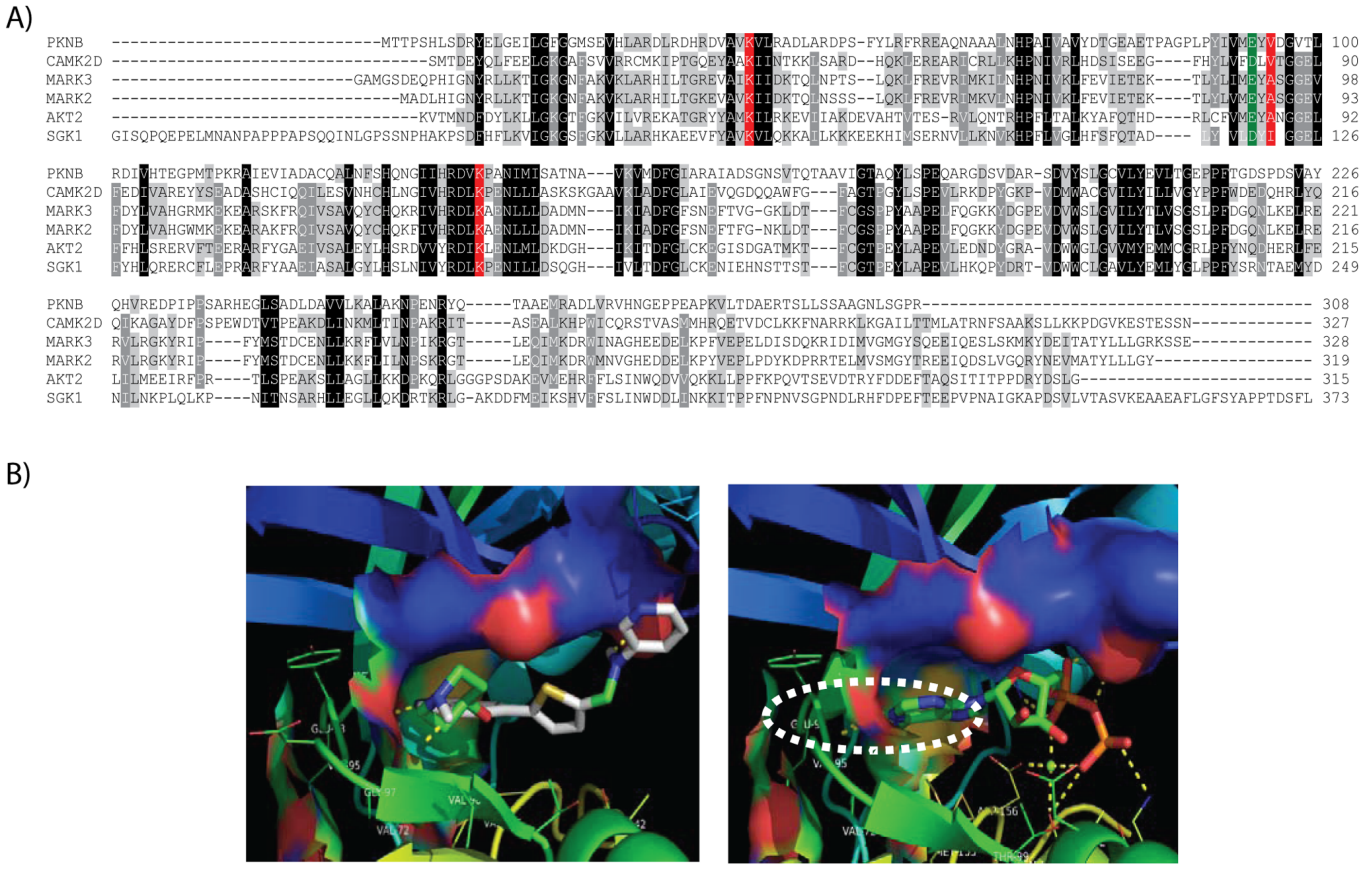
PknB kinase docking to GSK1598164A. **A**) Multiple sequence alignment of *Mycobacterium* PknB kinase with selected human kinases. Human kinases were selected on the criteria of having available PDB structures and top Psi-BLAST scores to *M. bovis* transmembrane serine/threonine-protein kinase B (pknB). First sequence in the alignment (gene name; PDB identifier) is *M. tuberculosis* transmembrane serine/threonine-protein kinase B (PknB; 3F69), which is 99% identical to *M. bovis* PknB and was used in compound docking models. Other sequences are CAMK2D (2EWL), MARK3 (2QNJ), MARK2 (3IEC), AKT2 (1GZK) and SGK1 (2R5T). Residues known to interact with ADP in pknB are highlighted in red. The amino acids aligned with Glu93, which may be essential for the binding of the GSK1132084A, are highlighted in green. **B**) Binding models of the GSK1598164A and ADP within pknB binding site (left and right panels, respectively).

Even though target Rv0014c, a serine/threonine-protein kinase, was not identified as belonging to an enriched pathway (it is not annotated in the KEGG database), it was predicted by the HIST approach to be a target for the GSK1365028A, GSK1598164A, GSK275628A and GW664700A (all singleton families in our compound clustering). Kinases are the most prominent human target class having identifiable orthologs in both *M. tuberculosis* H37Rv and *M. bovis* BCG genomes (Figure 4A). The human genome encodes over 450 kinases, while *Mycobacterium* contains between 4 and 24 serine/threonine kinases, depending on the exact species (*M. tuberculosis* and *M. bovis* have 11 conserved kinases each). At least two of these kinases, pknA and pknB, have been determined to be essential for *in vitro* viability of *M. tuberculosis* [21]. To further evaluate potential MoA of kinase inhibitors, we computationally docked several compounds into the adenine-binding portion of the ATP binding pockets of the two available experimental structures for the essential kinase pknB. The criteria for choosing the compounds were whole cell screening activity of MIC90 less than 10 µM and IC50 less than 8 µM. Two structures (PDB IDs: 2PZI and 3F69) were selected because both were co-crystallized with an inhibitor, clearly detailing their ATP binding pockets.

An empirical docking score threshold of −8.5 kJ/mol was chosen to identify putative positive bindings of the active compounds across the two pknB PDB models (Table S2). GSK1598164A, an inhibitor of several human serine/threonine protein kinases, was positive in both H37RV and BCG whole cell screens, based on favorable docking scores (−9.19 and −8.96 kJ/mol against 2PZI and 3F69, respectively). Both GSK1598164A and the enzymatic product ADP in the crystal structure were found to interact with the Glu93 of pknB, where the nitrogen atoms on the ‘head’ unit form the hydrogen bond with Glu93 (Figure 4B). Glu93 is conserved across both human and TB kinases (Figure 4A). Several residues in the putative hydrophobic binding pocket (Leu17, Gly18, Phe19, Val25, Ala38, Val72, Met92, Glu93 and Val95) were also found to be within 4 Å of both GSK1598164A and ADP. In conclusion, our analysis suggests that several bactericidal compounds in the published phenotypic screen act by inhibiting essential *M. tuberculosis* kinases.

### An example of a compound targeting the aminoacyl-tRNA biosynthesis pathway

The CHEM and STR methods identified Rv3598c (lysS1 lysine-tRNA ligase 1) and Rv3834c (serS serine-tRNA ligase) as possible targets for the GSK1402290A compound, respectively. Both enzymes are part of the aminoacyl-tRNA biosynthesis pathway (mtu00970) and are essential in *in vitro* experiments [21]. Moreover, the mtu00970 pathway was selected in our analysis as being significantly associated with GSKFAM_173 (GSK1402290A compound).

**Figure 5:**
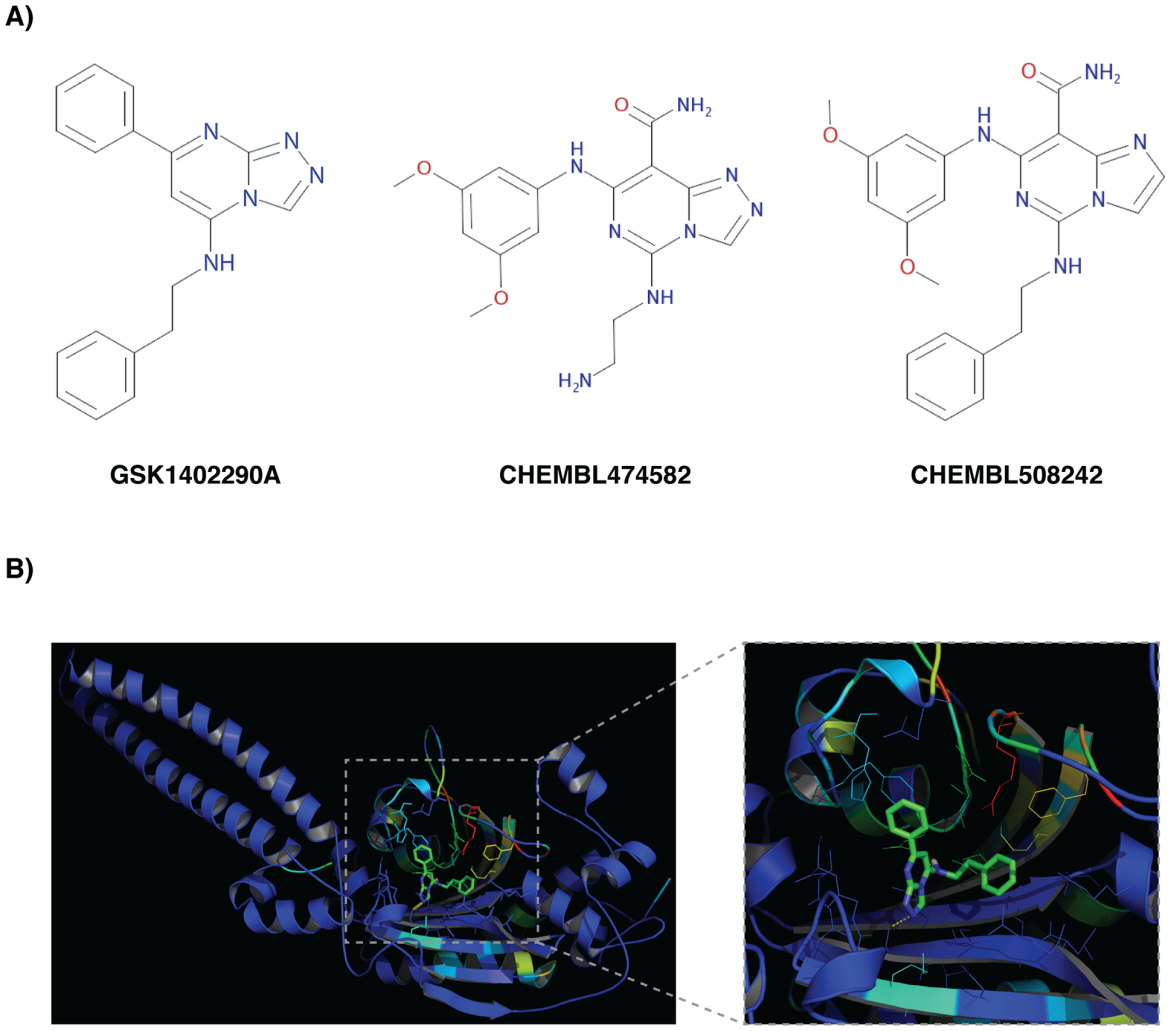
Targeting the aminoacyl-tRNA biosynthesis pathway. **A**) CHEM results show that GSK1402290A shared several substructural features with compounds reported as potent lysyl-tRNA synthetase inhibitors in the ChEMBL database (*e.g.*, CHEMBL474582 and CHEMBL508242). **B**) STR results predicted the serS as a target of GSK1402290A with its binding site including residues F205, H209, G225, T226, E228, R257, F276, K278, and E280, which are conserved in the PFAM family PF00587 (tRNA synthetase class II core domain). Zoomed image shows the pose for GSK1402290A predicted by AutoDock and the binding site residues (i.e., within 6 Å from the compound) coloured from low sequence conservation (blue) to high sequence conservation (red).

The CHEM approach predicted that the human lysyl-tRNA synthetase (UniProt ID Q15046) was a likely target of GSK1402290A, with a likelihood score of 11.3 and a Z-score of 2.4. Furthermore, the model indicated that the individual fragments contributing to this prediction were derived by its fused triazole ring (*e.g.*, pyrazole and imidazole features), as well as by its aniline group. In fact, the model for this target was trained using 47 active compounds from ChEMBL and almost all of them contained the aforementioned fragments (Figure 5A). Moreover, the predicted human target shared in OrthoMCL [24] the ortholog group (OG5_126972) with MTB's lysine-tRNA ligase 1 (UniProt ID P67607).

The STR method predicted a link between the compound and the target through a 3D model of the Rv3834c protein built based on the known structure of a seryl-tRNA synthetase from *Aquifex aeolicus*. The Rv3834c target and the seryl-tRNA synthetase template aligned with 43% sequence identity and resulted in good quality models (MPQS>1.5) [25]. To further evaluate potential MoA of the GSK1402290A compound, we computationally docked it into the nAnnoLyze predicted binding site for Rv3834c (Figure 5). The AutoDock run resulted in a best pose with −8,4 kJ/mol, indicating interactions between the GSK1402290A compound and the Rv3834c target (Figure 5B). In support of this model, the interactions occur with conserved protein residues, given the curated multiple sequence alignment for PFAM family PF00587 (tRNA synthetase class II core domain).

In summary, our CHEM and STR predictions suggest that GSK1402290A could act as an inhibitor of the aminoacyl-tRNA biosynthesis pathway and provide the basis for further chemical optimization of this compound.

### Open targets against tuberculosis

The recent publication of a large-scale screening effort for identifying drug-like small molecule compounds active against tuberculosis has been used as starting point for our research. Here, we predicted the likely mode of action of a selected set of compounds active against tuberculosis, based on a computational approach that integrates data from historical assay results, chemical features and their relationship to activity, and structural comparisons. Our integrated approach resulted in prediction of several compound-target pairs, which can be further tested using genomics, genetics and biochemical assays. More broadly, our approach can be applied to whole cell screens for any pathogen, provided sufficient datasets are available.

We have predicted a wide range of MTB specific as well as more evolutionary conserved targets. While compounds with known activity against a human protein could be compromised by toxicity, and therefore should be eliminated from further study, empirical evidence suggests that existence of a human orthologous sequences is not a strong filter for selecting pathogen targets. Many clinically used antibiotics have targets with human orthologs, such quinolones (DNA gyrase and topoisomerases), rifampicin (RNA polymerase), mupirocin (isoleucyl-tRNA synthetase) and the latest anti-TB drug now in Phase II testing, bedaquiline (F_1_F_0_ ATPase) [4], [6]. The associated side effects of antibiotics are mostly due to high doses treatments affecting off-target proteins (including human ortologs) and not specifically to on-target effects. The billion plus years of evolutionary distance between prokaryotes and mammals has lead to significant divergence between orthologous proteins such that there is sufficient structure activity relationship or SAR bandwidth to develop specific inhibitors of the pathogen target, in our case MTB.

It is important to note that we also had a subset of compounds with historical data indicating activity against human protein targets with no known homologs in MTB, such as the GPCRs. Thus, their mechanism of action against MTB must be due to non-human target related interactions. These compounds must be pursued with caution as drug candidates given their known *in vitro* interaction with a human protein. Nevertheless, such compounds could be valuable tools for understanding MTB viability. In general, knowledge of potential human protein interactions adds to the design of effective counter-screens to drive compound SAR specificity and potency towards the pathogen.

The public availability of the data and compounds [7] as well as our predictions (http://www.tropicaldisaes.org/TCAMSTB/ or ftp://ftp.ebi.ac.uk/pub/databases/chembl/tb) will facilitate further research on drug discovery against tuberculosis. A major goal of our work is to encourage other researchers to experimentally validate the described targets and make their findings publicly available as soon as possible, thus optimizing the process of developing a safe and well tolerated novel therapy for tuberculosis.

## Methods

### Compound dataset

All compound datasets used in this study (that is, BCG dataset of 776 GSK compounds including the H37Rv sub-dataset of 177 compounds) were obtained directly from the ChEMBL database (as deposition set http://dx.doi.org/10.6019/CHEMBL2095176). Chemical properties of the compounds (Figure 1) were calculated as previously described [12].

### Exploring the chemogenomics space

A multi-category Naïve Bayesian classifier (MCNBC) was built using structural and bioactivity information from the ChEMBL database (version 14) [16]. In brief, the classifier learns the various classes (in this case protein targets) by considering the frequency of occurrence of certain sub-structural features for the different chemical compounds. Given a new, unseen compound, the model calculates a Bayesian probability score based on the molecule's individual features and produces a ranked list of likely targets. The model was built in Accelrys Pipeline Pilot (version 8.5). The structure and bioactivity data were extracted from the ChEMBL database and conformed the following filters: (i) the activity value was better than 10 uM (pIC50>5), (ii) the target type was a protein, (iii) the activity type was IC50, Ki or EC50, and (iv) the target confidence score was above 7.0. The last filter ensured that there was a reported direct interaction between the ligand and the protein target. The script resulted in 489,056 distinct compound-target pairs. To increase the robustness of the model, only targets with 40 or more active compounds were considered further, thus reducing the number of unique compound-target pairs to 466,686, spanning 1,258 distinct targets and 271,918 distinct compounds.

Two multiple-category models were subsequently built. Firstly, a model was created by choosing at random 85% of the compound records as the training set, so that the remaining 15% could be used as a test set for model validation, ensuring no overlapping structures in the 85-15 partition [17]. The MCNBC trained on 85% of the 271,918 ChEMBL compounds and associated targets was then used to predict the targets for the remaining 15% of the ChEMBL subset, containing 40,788 distinct compounds, unseen by the model. Standard ECFP_6 fingerprints were employed as molecular descriptors for the classifier [26]. These fingerprints encode a molecular structure as a series of overlapping features/fragments of a diameter of up to three bond lengths.

For each compound in the test set, the Pipeline Pilot model generated a likelihood score *P_total_* for all possible targets. This is derived by the Laplacian-corrected Bayes rule of conditional probability *P(A|F_i_)* for each fingerprint feature i of the compound.
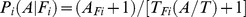


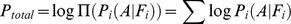
where *F_i_* is the *i* ^th^ fingerprint feature; *A* is the number of active molecules for a target; *T* is the total number of molecules; *A_Fi_* is the number of *active* molecules containing feature *i*; and *T_Fi_* is the number of all molecules containing feature *i*.

For the purposes of this validation, only the top five target predictions were considered (*i.e.*, the ones with the highest positive likelihood score). This reflects a real-life situation where only a small number of target predictions can be practically and economically tested experimentally. To test the accuracy of the method, the five target predictions were then compared to the actual target reported for that particular compound.

**Figure 6:**
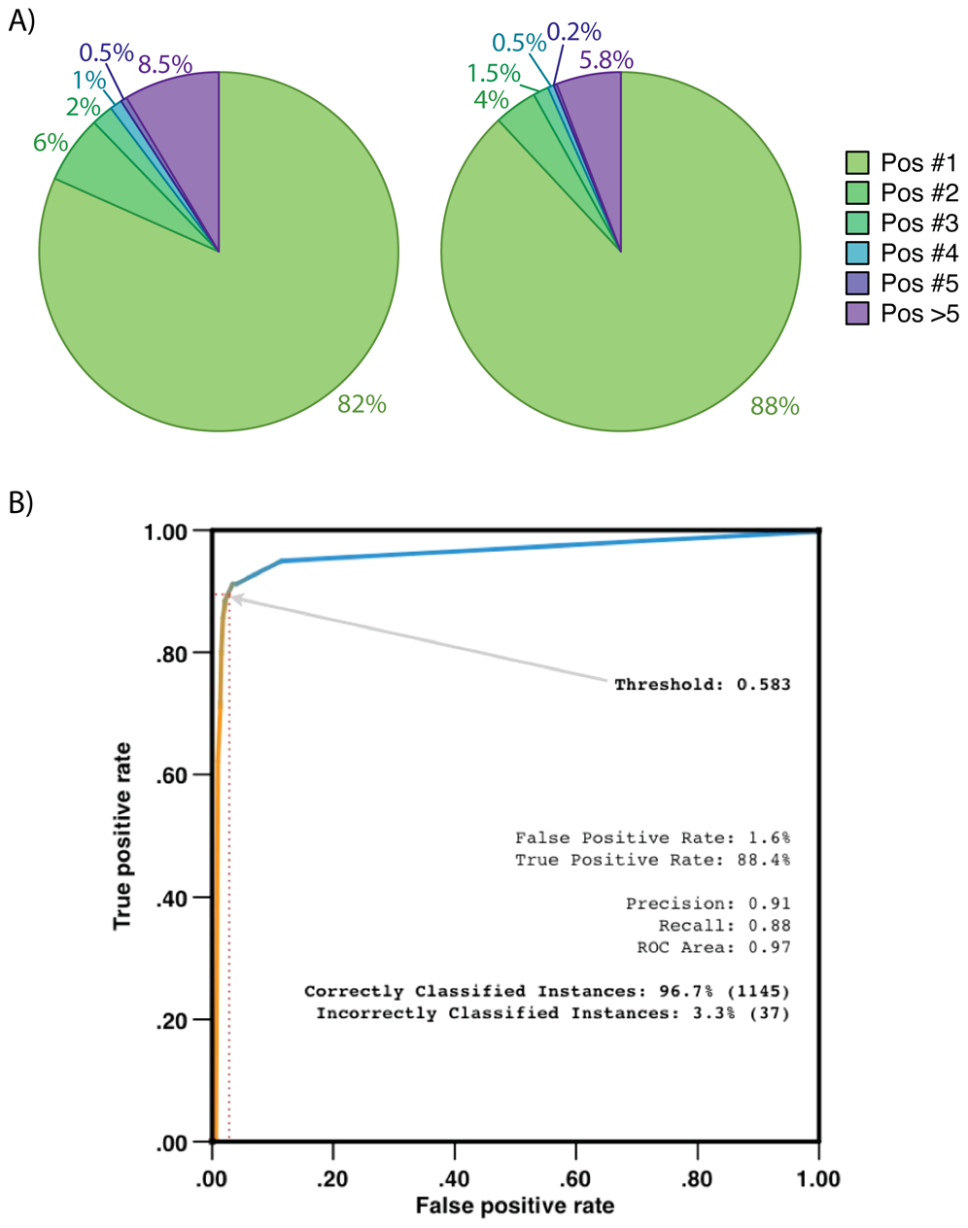
Predictive accuracy of the CHEM and STR methods. **A**) Predictive power of the MCNBC model using individual targets (left) or target classification information (right). **B**) Accuracy of the RFS differentiating similar from non-similar pairs of ligands. ROC curve indicates the optimal threshold for the RFS score of 0.58, which results in an area under the curve of 0.97 and a false positive rate of only 1.6%.

The model derived by the training set ranked the correct target highest among all 1,258 possible targets for 82% of the compounds in the test set (Figure 6A). The target is correctly predicted on the second guess for 6% of the compounds and correctly predicted on the third guess for 2% of the compounds. In total, 92% of the compounds in the test set are correctly assigned to their known targets within the top five predicted targets. The ChEMBL database groups most of the individual protein targets into a hierarchical classification of target family names. Given this information, further analysis was done to examine the accuracy of the target classification predictions. Individual targets were replaced by their respective protein classification annotation using a lookup dictionary. In total, 568 unique protein classification labels were considered. The model's predictive power improves, returning the correct protein family as the top ranked prediction in 88% of the compounds and within the top five predictions in 94% of the compounds (Figure 6A). After the successful validation of the method, a second model was created utilizing 100% of the data and keeping the rest of the parameters intact. The derived model was then used for predicting the targets of all GSK compounds.

### Exploring the structural space

A network of structural similarities between compounds and targets was built to identify the most likely target of a given compounds in our GSK dataset. To explore the structural space we used an improved version of our previously published AnnoLyze algorithm [14], which was based on homology detection through structural superimposition of targets and their interaction networks to small compounds similarly to previously published approaches [27], [28]. Briefly, the new nAnnoLyze algorithm relies in four pre-built layers of interconnected networks, First, the “GSK Ligand” network where nodes are GSK compounds and edges correspond to their similarity as measured by a Random Forest classifier score (RFS) (see below). Second, the “PDB Ligand” network where nodes are ligands in the Protein Data Bank (PDB) [29] and edges correspond to their similarity also measured by the RFS. The “GSK Ligand” network is linked to the “PDB ligand” network by edges corresponding to the compound similarity measure by the RFS. Third, the “PDB Protein” network where nodes are proteins in PDB and edges corresponds to their structural similarity as measured with the MAMMOTH structural superimposition [30]. Fourth, the “MTB Models” network where nodes are structure models of MTB targets and edges corresponds to their structural similarity after superimposition by the MAMMOTH program. The two central networks (that is, “PDB Ligand” and “PDB Protein” networks) are connected by co-appearance in any solved structure in the PDB and the “PDB Protein” and the “MTB Models” networks are also linked by the structural comparison between any protein in the PDB and all models from MTB. Finally, once all the networks are constructed, we identified the closest path between any GSK compounds and a MTB target and scored their relationship as the sum of all similarities scores in the network. Such score was then normalized between 0 (non-similar) and 1 (similar) and only pairs of GSK compounds and their MTB targets with scores higher than 0.4 were kept.

To identify whether two compounds could be considered similar, we developed a new Random Forest classifier (RFS), which was trained with a dataset of “similar” and “non-similar” ligands. Two ligands were similar if they bind the same binding site as defined by the LigASite database, a gold-standard dataset of biologically relevant binding sites in protein structures [31]. To avoid overestimation in the validation of our approach, all ligands in the database that were included in a testing set of 2,380 ligands from the PDB were removed. Our training set of similar ligands included 197 pairwise comparisons considered as “true similar” and a set of randomly paired ligands as “true non-similar” comparisons. The SMSD program [32] was then used to compare all pair of selected ligands to obtain their Tanimoto score, bond breaking energy, Euclidian distance for equivalent atoms, stereochemical match, substructure fragment size, and finally the molecular weight difference. Such scores were then normalized and constituted a vector defining the similarity between any two compared ligands, which was then used as input for the Random Forest classifier. The aim of the classifier was thus to identify hidden relationships between the six scores to maximize its capacity to identify true pairs of similar ligands and discern them from non-similar ligands. The classifier was then tested with a 10-fold cross validation procedure and resulted in an area under the ROC curve of 0.97 and a very small false positive rate of 1.6% (Figure 6B).

To populate the “MTB Model” network with structures of MTB targets, we built all possible comparative structure models for any protein in the *M. tuberculosis* H37Rv, *M. bovis* BCG, and *M. smegmatis* genomes using the ModPipe program [25]. All sequences were obtained from the Genomes Web site of the NCBI database. Such modeling resulted in a total of 34,894 comparative models for which 5,008 were predicted to be reliable models (that is, 1.1 or higher ModPipe quality score and ga341 higher than 0.7). Next, we structurally compared this set of selected models to any non-redundant (90% sequence identity) structure in the PDB that contained at least one known ligand. Structural comparisons between two proteins were performed using the MAMMOTH algorithm [30]. Four different scores were stored for each structural superimposition: percentage of sequence and structure identity for the entire protein and percentage of sequence and structure identity for the residues involved in the binding site defined as any residue in the PDB template structure within 6 Ångstroms of any atom in the ligand. A binding site in a model was considered then similar to a binding site in a known PDB structure if at least the binding site sequence and structure similarity were higher than 40%. This similarity cut-off was previously validated in a large-scale comparison of known ligand-protein pairs [14].

The final entire network of comparisons included the 776 compounds from the GSK dataset, ∼2.500 unique ligands from the PDB, ∼16,000 unique protein structures from the PDB and a total of ∼5,000 structure models from MTB. Such network resulted in 207 pairs of GSK compound to MTB target short paths (*i.e.*, score >0.4).

### Exploring historical assay data

GSK proprietary compound screening databases were queried for any historical assay data associated with both *Mycobacterium* species active compounds. The majority of these screens were against human protein targets. The threshold above which compound efficacy against specific human targets was considered significant was defined as pIC50≥5.0 for inhibition or antagonist assays, and pEC50≥5.0 for agonist, activation or modulator assays. Activities at more than 600 target-result type combinations (some targets are assayed in both an antagonist and agonist mode) were analyzed amongst the BCG and H37Rv active compounds, representing potential modes of action. The target activities for the screened compounds were analyzed to identify targets over-represented amongst the anti-malarial actives vs. inactives.

Using BLASTP [33] we queried the protein complement of published MTB H37Rv and *M. bovis* BCG genomes with RefSeq proteins [34] for all human targets accepting a homology cut-off of an E-value ≤1.0e-10 and visual inspection of the alignments. Putative homologous relationships were confirmed by reciprocal BLASTP searches of identified *Mycobacterium* homologues against the human RefSeq protein databases. Initial multiple sequence alignments were performed using the program CLUSTALW v1.8 [35] with default settings and subsequently refined manually using the program SEQLAB of the GCG Wisconsin Package v11.0 software package (Accelrys, San Diego, CA, USA).

### Statistical assessment of predicted links between compounds and targets

We measured two different statistics to assess the significance of a particular link between a chemical compound and a target pathway. Firstly, we calculated the LogOdds (that is, the odds of an observation given its probability). A feature *i* (in our case, a compound in Figure 2C or a pathway in Figure 3B) has a probability (*p_i,c_*) in the entire dataset and a probability (*p_i,r_*) of being at the subset of selected compounds/pathways. Their LogOdds are defined as the logarithm of its Odds (*O_i_*):
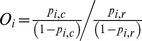
Therefore, Odds higher than 1 (or positive LogOdds) indicate over-occurrence of the compound/pathway in the selected subset. Odds smaller than 1 (or negative LogOdds) indicate under-representation of the compound/pathway in the selected subset. Secondly, a *p-value* score was calculated for each predicted link between a compound and a target pathway using a Fisher's exact test for 2×2 contingency tables comparing two groups of annotations (*i.e.*, the group of compounds in a given pathway and the group of compounds in the entire dataset) [36].

### Computational docking of compound in the structure of selected targets

Autodock 4.2 was used for docking studies [37]. The *ga_num_evals* were set at 250,000 to balance docking performance and CPU consumption. Thirty replicates were run for each chemical-protein pair and the binding conformation with the lowest docking score was chosen for visualization using PyMOL.

## Supporting Information

Figure S1 **Target class space.** A) For positive hits in *M. tuberculosis* H37Rv screens, the distribution of human target classes affected by compounds based on known human protein potency and selectivity criteria as described in the text. The number of human targets is indicated for each class as well as the potential number of homologous genes (in parentheses). B) Distribution of 49 compounds screened against 1 or more targets having pIC50 or pEC50 values >5.5 in 120 assays by human target classes. Some compounds have historical assay information and potency against multiple target classes. Also indicated is the number of assays against targets with putative homologues in *M. tuberculosis* (in parentheses). C) Similar analysis of human target classes and D) 240 compounds in 642 assays for *M. bovis* BCG screens.
(DOCX)

Table S1 **Predicted M. tuberculosis H37Rv and M. bovis BCG gene targets based on homology to human target assays.**
(DOCX)

Table S2 **Docking scores for the active compounds across two pknB structure models.**
(DOCX)
