# Supplementary material for: Target Prediction for an Open Access Set of Compounds Active against Mycobacterium tuberculosis
Source: PLoS Comput Biol. 2013 Oct 3;9(10):e1003253. doi: 10.1371/journal.pcbi.1003253 (PMC3789770; doi:10.1371/journal.pcbi.1003253)
Supplement: Figure S1 — Target class space. A) For positive hits in M. tuberculosis H37Rv screens, the distribution of human target classes affected by compounds based on known human protein potency and selectivity criteria as described in the text. The number of human targets is indicated for each class as well as the potential number of homologous genes (in parentheses). B) Distribution of 49 compounds screened against 1 or more targets having pIC50 or pEC50 values >5.5 in 120 assays by human target classes. Some compounds have historical assay information and potency against multiple target classes. Also indicated is the number of assays against targets with putative homologues in M. tuberculosis (in parentheses). C) Similar analysis of human target classes and D) 240 compounds in 642 assays for M. bovis BCG screens. (DOCX) [file pcbi.1003253.s001.docx]

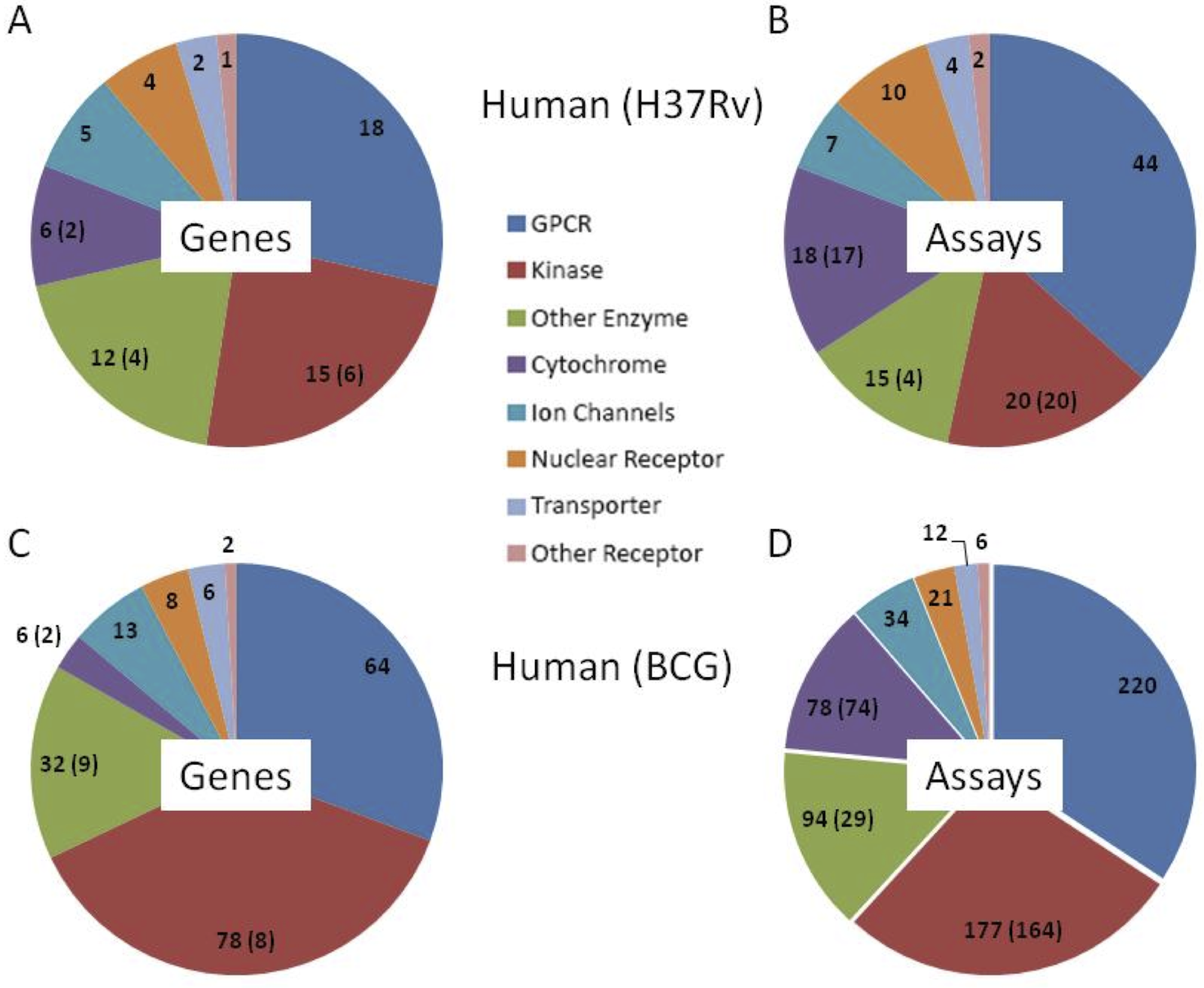


**Figure S1. Target class space.** A) For positive hits in *M. tuberculosis* H37Rv screens, the distribution of human target classes affected by compounds based on known human protein potency and selectivity criteria as described in the text. The number of human targets is indicated for each class as well as the potential number of homologous genes (in parentheses). B) Distribution of 49 compounds screened against 1 or more targets having pIC50 or pEC50 values > 5.5 in 120 assays by human target classes. Some compounds have historical assay information and potency against multiple target classes. Also indicated is the number of assays against targets with putative homologues in *M. tuberculosis* (in parentheses). C) Similar analysis of human target classes and D) 240 compounds in 642 assays for *M. bovis* BCG screens.
