# Supplementary material for: Target Prediction for an Open Access Set of Compounds Active against Mycobacterium tuberculosis
Source: PLoS Comput Biol. 2013 Oct 3;9(10):e1003253. doi: 10.1371/journal.pcbi.1003253 (PMC3789770; doi:10.1371/journal.pcbi.1003253)
Supplement: Table S1 — Predicted M. tuberculosis H37Rv and M. bovis BCG gene targets based on homology to human target assays. (DOCX) [file pcbi.1003253.s002.docx]

**Table S1.** Predicted *M. tuberculosis* H37Rv and *M. bovis* BCG gene targets based on homology to human target assays

| Human Target Class | No. of Compounds | | Putative *Mycobacterium* Target | | Accession No. | | Essentiality |
| --- | --- | --- | --- | --- | --- | --- | --- |
|  | BCG | H37Rv | Gene | Product | M. bovis BCG | MTB H37Rv |  |
| Kinase | 35 | 8 | pknA | transmembrane serine/threonine-protein kinase A | YP_976148.1 | NP_214529.1 | Essential |
|  |  |  | pknB | transmembrane serine/threonine-protein kinase B | YP_976147.1 | NP_214528.1 | Essential |
|  |  |  | pknD | Ser/Thr protein kinase | YP_977078.1 | NP_215446.1 | NE |
|  |  |  | pknH | putative transmembrane serine/threonine-protein kinase H | YP_977417.1 | NP_215782.1 | NE |
|  |  |  | pknJ | putative transmembrane serine/threonine-protein kinase J | YP_978197.1 | NP_216604.1 | NE |
|  |  |  | pknL | putative transmembrane serine/threonine-protein kinase L | YP_978280.1 | NP_216692.1 | NE |
|  |  | None | pknF | anchored-membrane serine/threonine-protein kinase F | YP_977877.1 | NP_216262.1 | NE |
|  |  | None | pknK | putative serine/threonine-protein kinase transcriptional regulatory protein K | YP_979189.1 | NP_217596.1 | NE |
| Other Enzyme | 1 | 0 | aao | Putative D-amino acid oxidase | YP_978034.1 |  | NE |
|  | 5 | 2 | amiB2 | amidase | YP_977414.1 | NP_215779.1 | NE |
|  | 2 | 1 | aofH | putative flavin-containing monoamine oxidase | YP_979728.1 | NP_217686.1 | NE |
|  | 2 | 0 | BCG_1212c | NAD-dependent deacetylase | YP_977305.1 |  | NE |
|  | 1 | 0 | BCG_1741c | Putative catechol-O-methyltransferase | YP_977833.1 |  | NE |
|  | 9 | 1 | mycP1 | Protease | YP_980017.1 | NP_218400.1 | NE |
|  | 1 | 0 | ephB | Putative epoxide hydrolase | YP_978066.1 |  | NE |
|  | 2 | 0 | fabG | 3-ketoacyl-(acyl-carrier-protein) reductase | YP_977504.1 |  | Essential |
|  | 3 | 1 | pyrD | Dihydroorotate dehydrogenase 2 | YP_978245.1 | NP_216655.1 | NE |
| Cytochrome | 49 | 13 | cyp132 | putative cytochrome P450 132 | YP_977547.1 | YP_177807.1 | NE |
|  |  |  | cyp136 | putative cytochrome P450 136 | YP_979168.1 | NP_217575.1 | NE |
| GPCR | 111 | 24 | No homolog |  |  |  |  |
| Ion Channel | 31 | 6 | No homolog |  |  |  |  |
| Nuclear Receptor | 20 | 9 | No homolog |  |  |  |  |
| Other Receptor | 6 | 2 | No homolog |  |  |  |  |
| Other Enzyme | 3 | 8 | No homolog |  |  |  |  |
| Transporter | 9 | 4 | No homolog |  |  |  |  |
| Total | 180 | 54 |  |  |  |  |  |

*M. tuberculosis* H37Rv and *M. bovis* BCG homologs determined by BLASTP [[1](#_ENREF_1)] searches using human targets.

Human target classes are defined in the text. Some compounds may be active across more than one target class.

Essentiality scoring based on [[2](#_ENREF_2)]. NE = No Evidence from these sources.

**References**

1. Altschul SF, Madden TL, Schaffer AA, Zhang J, Zhang Z, et al. (1997) Gapped BLAST and PSI-BLAST: a new generation of protein database search programs. Nucleic Acids Res 25: 3389-3402.

2. Sassetti CM, Boyd DH, Rubin EJ (2003) Genes required for mycobacterial growth defined by high density mutagenesis. Mol Microbiol 48: 77-84.
