# Supplementary material for: Target Prediction for an Open Access Set of Compounds Active against Mycobacterium tuberculosis
Source: PLoS Comput Biol. 2013 Oct 3;9(10):e1003253. doi: 10.1371/journal.pcbi.1003253 (PMC3789770; doi:10.1371/journal.pcbi.1003253)
Supplement: Table S2 — Docking scores for the active compounds across two pknB structure models. (DOCX) [file pcbi.1003253.s003.docx]

**Table S2.** Docking scores for the active compounds across two pknB structure models

| GSK number | pIC50 | Gene | Score_2PZI_* | Score_3F69_* |
| --- | --- | --- | --- | --- |
| GW623128X | 7.3 | *CNR2* | -8.83 | -8.63 |
| GSK1519001A | 5.7 | *NPSR1* | -9.09 | -8.65 |
| GSK547481A | 6.1 | *HTR4* | -8.93 | -9.27 |
| GSK2043267A | 7.5 | *CYP2C19* | -8.8 | -8.56 |
| GSK381407A | 5.6 | *P2RY14* | -9.02 | -8.71 |
| GSK547543A | 6.2 | *GPR55* | -9.03 | -10.37 |
| GSK547511A | 6.2 | *GPR55* | -9.01 | -8.58 |
| GSK1598164A | 8 | *IKBKB* | -9.19 | -8.96 |
| GSK1635139A | 5.6 | *CHRNA7* | -8.6 | -9.59 |

*2PZI and 3F69 are PDB IDs for the two publicly available experimental structure models for pknB
